# Bœnninghausen’s Treatment of Croup

**Published:** 1889-03

**Authors:** A. McNeil


### "BŒNNINGHAUSEN'S TREATMENT OF CROUP."

I can scarcely nerve myself to the task of criticising such men as Bœnninghausen, Carroll Dunham, and P. P. Wells. They are men whom no praise or blame from me can belittle. And I yield to none in my admiration of them. But it is only in defense of principles that I can even appear to differ from such illustrious men.

My objections to the prescribing of Bœnninghausen's powders are, 1st. It is treating a disease or for a name, not patients, thereby entirely ignoring treating the totality of the symptoms. 2d. It is an alternation of remedies, and 3d. It is not the most successful way of curing croup. It is a work of supererogation for me to prove that the first two are not the correct way of treating disease to the readers of THE HOMŒOPATHIC PHYSICIAN. And, moreover, it is to Bœnninghausen, Dunham, and Wells that the best expositions of the error of thus treating disease that we are indebted. I refer to vol. V, page 529, of the *American Homœopathic Review* for a masterly attack on the alternation of remedies, by Dunham. As for Bœnninghausen, he discusses all such points in his invaluable work, which has not been translated, *Aphorismen des Hippokrates*, and the readers of THE HOMŒOPATHIC PHYSICIAN need not be reminded of the ability with which the venerable P. P. Wells defends the fundamental principles of Homœopathy. I may, therefore, confine my attention to the last proposition, viz., that this un-Hahnemannian prescribing is not successful in curing croup or anything else. Permit me here to give my own experience in the treatment of croup. During the first five years of my professional life croup was my horror. Speaking from recollection, I think that half of my croup patients died. I used Bœnninghausen's five powders repeatedly, and I gave those drugs separately. I gave all remedies for croup that were recommended by those I considered reliable, and I used the different medicines, mostly in medium potencies (30th), and the result was the same. I assert that I never saw any good come from Aconite. I recall one case that I cured with Spongia alone. After five years of such practice, I worked my way up to prescribing homœopathically, viz., according to the precepts of Hahnemann. And in fifteen years I have not lost a patient that had not had another physician first. I had one die in San Francisco who had been given up by another.

What produced the change in results? A close following of the principles of Hahnemann, viz., prescribing for the patient and not the disease. I stopped all giving of "croup" remedies, and gave the simillimum, usually in the 30th or 200th, and as soon as improvement was perceptible, gave placebo. Speaking again from recollection, I cured with Mercurius, Tartar emetic, Lachesis, Bryonia, and Rhus tox., given according to the totality of the symptoms, and the remedy given was generally the one corresponding to the *genus epidemicus*.

But the question may arise in the minds of my hearers, Did Bœnninghausen, Dunham, and Wells give false reports when they relate such brilliant results? By no means. I have too great a reverence for those illustrious men to whom I am so much indebted, to even think it possible. I cannot turn to the passage of Bœnninghausen's works in which he gave those instructions, but I am of opinion that they were intended for the laity, and as many of the believers of Homœopathy in Germany then, and even now, would not avail themselves of the aid of good homœopathic physicians such was justifiable, and I also believe that at that time in Germany, and when Dunham and Wells had such results in the United States, Aconite was the epidemic remedy and Hepar followed it well, owing to the relation in which these drugs stand to each other, and for the same reason the Spongia did well after Hepar had done its work. I am confident now that, in San Francisco at least, Bryonia and Rhus given in that way would have caused equally as brilliant results as those celebrated physicians have recorded. And at no time has Aconite been the epidemic remedy since I began the practice of medicine. As to why Aconite, Hepar, and Spongia should make so many cures of a disease at one time and other drugs at another time, would require more space than the genial editor of THE HOMŒOPATHIC PHYSICIAN would be willing to grant or my kind readers time to read in addition to this long article. A. McNEIL, M. D.

